# Extracellular Vesicles from *Scedosporium apiospermum* Mycelial Cells: Implication for Fungal-Host Interplays

**DOI:** 10.3390/jof10040277

**Published:** 2024-04-09

**Authors:** Ana Carolina Aor, Leandro S. Sangenito, Thaís P. Mello, Luna S. Joffe, Juliana Rizzo, Venício F. Veiga, Renata N. da Silva, Marcos D. Pereira, Beatriz B. Fonseca, Sonia Rozental, Rosa Maria T. Haido, Marcio L. Rodrigues, Marta H. Branquinha, André L. S. Santos

**Affiliations:** 1 Departamento de Microbiologia Geral, Instituto de Microbiologia Paulo de Góes (IMPG), Centro de Ciências da Saúde (CCS), Universidade Federal do Rio de Janeiro (UFRJ), Rio de Janeiro 21941-902, RJ, Brazillujoffe@gmail.com (L.S.J.); veigavf@gmail.com (V.F.V.); marciolrodrig@gmail.com (M.L.R.); mbranquinha@micro.ufrj.br (M.H.B.); 2 Departamento de Microbiologia e Parasitologia (MIP), Instituto Biomédico (CMB), Universidade Federal Fluminense (UFF), Niterói 24210-130, RJ, Brazil; 3 Instituto Federal de Educação, Ciência e Tecnologia do Rio de Janeiro (IFRJ), Campus Nilópolis, Rio de Janeiro 26530-060, RJ, Brazil; 4 Instituto de Biofísica Carlos Chagas Filho (IBCCF), Centro de Ciências da Saúde (CCS), Universidade Federal do Rio de Janeiro (UFRJ), Rio de Janeiro 21941-170, RJ, Brazilrozental@biof.ufrj.br (S.R.); 5 Programa de Pós-Graduação em Bioquímica, Instituto de Química, Universidade Federal do Rio de Janeiro (UFRJ), Rio de Janeiro 21941-909, RJ, Brazil; renatanascimento.94@hotmail.com (R.N.d.S.); marcosdpufrj@gmail.com (M.D.P.); 6 Rede Micologia RJ—Fundação de Amparo à Pesquisa do Estado do Rio de Janeiro (FAPERJ), Rio de Janeiro 21941-902, RJ, Brazil; 7 Departamento de Microbiologia e Parasitologia, Instituto Biomédico, Universidade Federal do Estado do Rio de Janeiro (UNIRIO), Rio de Janeiro 20211-010, RJ, Brazil; rmthaido@yahoo.com.br; 8 Instituto Carlos Chagas, Fundação Oswaldo Cruz (Fiocruz), Curitiba 81310-020, PR, Brazil

**Keywords:** *Scedosporium apiospermum*, extracellular vesicles, immunogenic molecules, cellular interaction, cytotoxicity, virulence

## Abstract

The release of extracellular vesicles (EVs) has been implicated as an alternative transport mechanism for the passage of macromolecules through the fungal cell wall, a phenomenon widely reported in yeasts but poorly explored in mycelial cells. In the present work, we have purified and characterized the EVs released by mycelia of the emerging, opportunistic, widespread and multidrug-resistant filamentous fungus *Scedosporium apiospermum*. Transmission electron microscopy images and light scattering measurements revealed the fungal EVs, which were observed individually or grouped with heterogeneous morphology, size and electron density. The mean diameter of the EVs, evaluated by the light scattering technique, was 179.7 nm. Overall, the structural stability of *S. apiospermum* EVs was preserved during incubation under various storage conditions. The lipid, carbohydrate and protein contents were quantified, and the EVs’ protein profile was evidenced by SDS-PAGE, revealing proteins with molecular masses ranging from 20 to 118 kDa. Through immunoblotting, ELISA and immunocytochemistry assays, antigenic molecules were evidenced in EVs using a polyclonal serum (called anti-secreted molecules) from a rabbit inoculated with conditioned cell-free supernatant obtained from *S. apiospermum* mycelial cells. By Western blotting, several antigenic proteins were identified. The ELISA assay confirmed that the anti-secreted molecules exhibited a positive reaction up to a serum dilution of 1:3200. Despite transporting immunogenic molecules, *S. apiospermum* EVs slightly induced an in vitro cytotoxicity effect after 48 h of contact with either macrophages or lung epithelial cells. Interestingly, the pretreatment of both mammalian cells with purified EVs significantly increased the association index with *S. apiospermum* conidia. Furthermore, EVs were highly toxic to *Galleria mellonella*, leading to larval death in a typically dose- and time-dependent manner. Collectively, the results represent the first report of detecting EVs in the *S. apiospermum* filamentous form, highlighting a possible implication in fungal pathogenesis.

## 1. Introduction

*Scedosporium apiospermum* is a saprophytic filamentous fungus that has emerged as an opportunistic pathogen, causing invasive fungal diseases in humans [1]. In healthy individuals, this fungus can cause subcutaneous infections, known as mycetoma, due to the traumatic inoculation of conidial cells and/or mycelial fragments or brain abscesses associated with aspiration of polluted water in cases of drowning [2,3]. In immunocompromised individuals, inhalation of conidia can lead to bronchopulmonary mycoses and pneumonia. Following pulmonary colonization, invasion of deeper tissues may occur, progressing to severe illnesses, such as endocarditis, aneurysms, cerebral abscesses and meningitis, conditions that are difficult to treat and exhibit mortality rates of up to 90% [4,5,6]. In addition, *Scedosporium* species are ranked as the second most commonly isolated genus from cystic fibrosis patients, just behind *Aspergillus fumigatus* [7]. Treatment options for scedosporiosis are extremely limited and have high rates of therapeutic failure due to the multidrug-resistance profile associated with this fungal genus [8,9]. Infections caused by *Scedosporium* spp. share similar characteristics with microbial biofilms, including a high density of mycelial cells enclosed in a self-produced extracellular matrix [10,11,12]. In vitro, the high resistance found in *Scedosporium* biofilms is due to a multitude of mechanisms that operate concurrently, including an increase in cell density, high activity of efflux pumps and the presence of an extracellular polymeric matrix that prevents drug diffusion to fungal cells within biofilms [11,12]. 

In view of this alarming scenario, further studies on the cellular biology and pathogenesis of this clinically relevant fungus are urgently needed. In this regard, research related to the secretion of extracellular molecules is particularly relevant for understanding fungal pathogenesis [13,14]. The literature on the detection and characterization of extracellularly released molecules by *S. apiospermum* includes few reports of antigenic molecules, molecules with cytotoxic activities, siderophores and hydrolytic enzymes, especially those belonging to the peptidase class [12,15,16,17]. 

In the last decade, not only has the composition of extracellular molecules attracted the attention of the scientific community, but also the way fungal molecules reach the extracellular environment. In fungi, molecules destined for the extracellular space must traverse a rigid and complex structure—the cell wall. Many proteins cross the cell wall and reach the extracellular environment within vesicular compartments called extracellular vesicles (EVs) [18]. EVs are produced by all living organisms. Fungal EVs exhibit diverse biological roles, such as regulating physiological features, responding to specific environmental conditions, and mediating interkingdom communications [19]. Moreover, EVs have been shown to carry (glyco)proteins, (glyco)lipids, polysaccharides, signaling molecules and RNA; some of these components are well-known virulence attributes described in fungi. Therefore, these reports support the hypothesis that EVs concentrate molecules that participate in the fungal pathogenic process [13,20,21,22,23,24]. 

The first report regarding the isolation and characterization of EVs in a filamentous fungus was in *Alternaria infectoria*, a ubiquitously environmental organism that rarely causes disease in humans or animals [25]. Currently, EVs have already been described in some other phytopathogenic filamentous fungi, such as *Fusarium oxysporum* f. sp. *vasinfectum*, *Fusarium graminearum*, *Zymoseptoria tritici*, *Ustilago maydis* and *Colletotrichum higginsianum* [26,27,28,29,30,31]. In relation to filamentous fungi of medical relevance, the data available so far are limited [19]. EVs were isolated and characterized from *Trichophyton interdigitale* [32], *Rhizopus delemar* [33], *Aspergillus flavus* [34] and *A. fumigatus* [35]. More recently, the first characterization of EVs in protoplasts obtained from germinating conidia of *A. fumigatus* was described, showing that EV production was a common feature of different morphological stages of this opportunistic fungal pathogen. In that study, proteomic and carbohydrate analyses revealed the presence of enzymes related to both lipid and sugar metabolisms, cell wall biosynthesis and relevant pathogenic events [14]. In 2012, our group suggested the presence of vesicle-like structures in *S. apiospermum* by transmission electron microscopy, which revealed the presence of vesicles close to and fused to the plasma membrane, as well as inside the cell wall and free in the extracellular medium [17]. 

In the present study, our primary aim was to isolate and characterize the EVs from *S. apiospermum* mycelial cells. Subsequently, we evaluated the influence of EVs on the in vitro interaction between *S. apiospermum* conidia and mammalian cells (macrophage and lung epithelial lineages). Finally, we tested the toxicity of *S. apiospermum* EVs in experiments using *G. mellonella* larvae as an in vivo model.

## 2. Materials and Methods

### 2.1. Fungal Growth Conditions

*Scedosporium apiospermum* (strain RKI07_0416) was kindly provided by Dr Bodo Wanke (Evandro Chagas Hospital, Fundação Oswaldo Cruz, Rio de Janeiro, Brazil). The fungus in its mycelial form was cultured in Erlenmeyer flasks containing 200 mL of Sabouraud-dextrose broth (2% glucose, 1% peptone and 0.5% yeast extract) at room temperature (RT), with constant shaking (200 rpm) for 7 days [36]. Subsequently, the mycelial cells were filtered using filter paper and washed twice with sterile phosphate-buffered saline (PBS; 10 mM NaH_2_PO_4_, 10 mM Na_2_HPO_4_, 150 mM NaCl, pH 7.2). To obtain conidial cells for the interaction assays, the fungus was grown on potato dextrose agar (PDA; Difco Laboratories, Franklin Lakes, NJ, USA) plates at RT for 7 days. Conidia were then harvested by washing the plate surfaces with PBS and filtering them through a 40 μm nylon cell strainer (BD Falcon, Saffron Walden, UK) to remove hyphal fragments [30]. The conidial cells were counted using a Neubauer chamber.

### 2.2. Fungal Secretion and Viability Assay

The *S. apiospermum* mycelial cells (~12 g) were resuspended in Erlenmeyer flasks containing 1000 mL of sterile isotonic PBS supplemented with 2% glucose [17]. The systems were incubated for 24 h at RT with constant agitation (200 rpm). After this interval, the mycelial cells were separated from the conditioned supernatant by vacuum filtration. To obtain the dry weight, the mycelia were autoclaved, dried at 60 °C for 10 h and weighed on an analytical balance. In parallel, the fungal-conditioned supernatant was passed through a 0.22 μm membrane with a cutoff of 100 kDa (Millipore, São Paulo, SP, Brazil). The cell-free conditioned supernatant was concentrated in a 10,000 molecular weight cutoff AMICON micropartition system (AMICON, Beverly, MA, USA), obtaining a final concentration of 50-fold and kept at −20 °C until used. To guarantee the viability of mycelia used in all the experimental conditions, calcofluor white and propidium iodide double staining was performed as previously described [17]. To conduct this, mycelial cells were incubated for 30 min at RT with calcofluor white (Sigma-Aldrich, St. Louis, MO, USA) at a concentration of 1 mg/mL. Subsequently, the cells were washed with PBS and incubated with propidium iodide at 10 μg/mL (Sigma-Aldrich) for 10 min. Afterward, the cells were washed again with PBS and immediately examined under a Zeiss epifluorescence microscope (Axioplan, Jena, Germany). Fungal cells killed by autoclaving (121 °C for 30 min) were used as positive controls for staining with propidium iodide [17]. 

### 2.3. Isolation of EVs

The concentrated cell-free conditioned supernatant was centrifuged twice at 19,000× *g* for 20 min at 4 °C to remove smaller debris. The pellet was discarded, and the resulting supernatant was then subjected to higher centrifugation at 100,000× *g* for 1 h at 4 °C. The supernatant was discarded, and the pellet was washed twice with PBS by sequential resuspension and centrifugation steps, each consisting of 100,000× *g* for 1 h at 4 °C [37]. Finally, the pellet corresponding to the purified EVs was resuspended in sterile PBS or Dulbecco’s modified Eagle’s medium (DMEM), filtered, kept on ice and immediately used in the experiments.

### 2.4. Detection of EVs

#### 2.4.1. Mycelium Observation

Transmission electron microscopy (TEM) analysis was initially performed to detect possible vesicles associated with the fungal structures. To conduct this, mycelial cells were fixed for 1 h at 25 °C in 100 mM cacodylate buffer (pH 7.4, containing 2.5% glutaraldehyde, 4% paraformaldehyde and 10 mM calcium chloride). After fixation, the mycelia were washed in cacodylate buffer and then postfixed with 1% OsO_4_ and 0.8% K_4_Fe(CN)_6_.3H_2_O in 100 mM cacodylate buffer for 2 h. Samples were then washed in cacodylate buffer, dehydrated in a graded series of acetone and embedded in Spurr resin. Ultrathin sections were routinely stained with aqueous uranyl acetate and alkaline lead citrate and examined in a TEM type JEOL 1200 EX (JEOL Ltd., Akishima, Japan), operating at 80 kV [38].

#### 2.4.2. EVs Observation

The purified EVs were fixed in 0.1 M cacodylate buffer for 24 h at 4 °C. Afterward, the samples were incubated for 90 min in 2% osmium tetroxide, dehydrated in series in acetone and soaked in EPON 812 resin. Ultrathin sections were obtained in ultramicrotome and placed in nickel grids coated with 0.4% Formvar film. The grids were contrasted with uranyl acetate for 30 min and then with lead citrate for 5 min. In parallel, for negative staining, the EVs were incubated with a 2% phosphotungstic acid solution, pH 7.2, for 1 min in a copper grid with 0.4% Formvar film. The samples were observed under a JEOL 1200 EX transmission electron microscope, operating at 80 kV [39].

### 2.5. Production of an Immune Serum against Secreted Molecules of S. apiospermum

An adult white male rabbit was inoculated with a suspension containing 2 mg/mL of fungal antigens (corresponding to the cell-free conditioned supernatant obtained after growth of *S. apiospermum* mycelial cells in PBS-glucose for 24 h at RT), emulsified in an equal volume of complete Freund’s adjuvant. Two intramuscular doses were administered with an interval of 1 month between them. A third dose was given subcutaneously, performed 3 weeks after the second dose. A booster dose, equal to the third dose, was administered 1 week before bleeding. Bleeding was performed by cardiac puncture, obtaining 20 mL of blood from the animal. The collected blood was incubated at 37 °C for 30 min and then incubated overnight at 4 °C for coagulation and clot retraction. The hyperimmune serum (called anti-secreted molecules) was separated by centrifugation and stored at −20 °C. A pre-immune serum was obtained before the first animal was challenged with fungal antigens [40]. The experiments were conducted following the guidelines of the Institutional Committee for Animal Care and Experimentation of the Federal University of the State of Rio de Janeiro (UNIRIO), Protocol CEUA-UNIRIO/2014.01.

### 2.6. SDS-PAGE and Western Blotting Analyzes 

Proteins from cell-free supernatant and EVs’ preparations were separated by electrophoresis in a Mini-Protean device (Bio-Rad, Hercules, CA, USA) [41]. The equivalent of 50 µg (cell-free culture supernatant) and 35 µg (EVs) of protein was dissolved in loading buffer (2% sodium dodecyl sulfate [SDS], 10% glycerol, 10 mM Tris-HCl [pH 6.8], β-mercaptoethanol 5% and bromophenol blue 0.0025%), heated at 100 °C for 5 min and separated by 12% polyacrylamide gel electrophoresis (PAGE) (120 V/120 mA). Prior to electrophoresis, Gibco BRL (Grand Island, NY, USA) molecular mass standards were boiled in SDS-PAGE sample buffer mentioned above and then applied to the same gel. The protein profile was visualized after impregnation with silver nitrate. In parallel, electrophoresed proteins were transferred to nitrocellulose membranes for 2 h (300 mA/120 V) in 12.2 mM Tris buffer and 96 mM glycine (pH 8.3) containing 10% methanol at 4 °C. After that, the membranes were blocked with 0.5% fat-free milk in PBS with 0.1% Tween 20 (PBS-T) for 1 h. Membranes were then incubated individually for 1 h at RT with the rabbit pre-immune serum (at 1:200 dilution) or the anti-secreted molecules (at 1:400 dilution). Subsequently, all the membranes were washed three times with PBS-T and incubated for 1 h with horseradish peroxidase (HRP)-conjugated anti-rabbit IgG (at 1:2500 dilution) (Sigma-Aldrich, Livonia, MN, USA). The chemiluminescent system ECL (Bio-Rad, Hercules, CA, USA) was used to develop the immunoblots [42]. 

### 2.7. Enzyme-Linked Immunosorbent Assay (ELISA)

A 96-well microtiter polystyrene plate was coated with 100 µL/well of antigen concentrations (20 µg/mL of supernatant or EVs) for 1 h at 37 °C, and then for 20 h at 4 °C in a humid chamber. Thereafter, 3 washes were performed with 0.05% PBS-T (150 µL/well). The non-specific sites were blocked by incubation with 0.1% PBS-T containing 5% skim milk (150 mL/well) (PBS-TM) for 1 h at 37 °C in a humid chamber, and then, 100 µL of the anti-secreted molecules (dilutions ranging from 1:100 to 1:6400) were incubated for 1 h at 37 °C. After washing the plates (5 times) with 0.1% PBS-T (150 µL/well), the anti-rabbit IgG antibody conjugated to HRP (Sigma-Aldrich, Livonia, MN, USA) was added (100 µL/well) and diluted to 1:2000 in PBS-TM, proceeding to incubation as in the previous step. Plates were washed and incubated at RT for 20 min in the dark with 100 μL of enzyme substrate (4 mg orthophenylenediamine in 10 μL of citrate buffer (pH 5.5) and 4 μL of 30 V of H_2_O_2_). The reaction was stopped with 50 μL of 3N H_2_SO_4_ and the absorbance was read at 492 nm. Pre-immune serum was used as a negative control [43].

### 2.8. Immunocytochemistry Assay

Mycelial cells were processed for immunocytochemistry in LR WHITE (Sigma-Aldrich, St. Louis, MO, USA) hydrophilic resin. The fungus was fixed for 24 h at 4 °C in a solution containing 0.2% glutaraldehyde, 4% formaldehyde, 0.1 M sodium cacodylate buffer, 3% sucrose, 5 mM calcium chloride and 0.2% picric acid. After that, the mycelial cells were washed several times in 15% ethanol for 5 min until complete removal of the fixation solution, followed by progressive dehydration in ethanol (30%, 50%, 70%, 90%, 100%) at RT for 15 min. Then, the process was continued through the infiltration step, where the samples were subjected to a battery of incubations of 24 h at RT in LR White/ethanol (*v*/*v*) (1:3; 1:2; 1:1; 2:1; 3:1), with the last incubation in pure LR White. After the last step, the solution was changed to a fresh one to begin the inclusion step where the mycelial pellet was introduced into the gelatin capsule to polymerize at 60 °C for 48 h. Ultrafine sections of the resin were obtained in ultramicrotome and placed in nickel grids to be incubated in 0.1 M Tris-buffered saline (TBS) and 50 mM ammonium chloride. After 3 washes in TBS, the grids were incubated in 50 mM ammonium chloride for 30 min and then blocked in two 15 min cycles in PBS containing 3% BSA (3% PBS-BSA), pH 8.0. After blocking, the grids were incubated for 1 h at RT with either anti-secreted molecules or rabbit pre-immune serum (negative control) [44] at 1:20 dilution and serial dilutions of 1:5 to 1:100 in PBS containing 1.5% BSA (1.5% PBS-BSA), pH 8.0, in a humid chamber. Subsequently, the grids were subjected to 3 successive washes of 10 min in 3% PBS-BSA. Then, the systems were incubated for 1 h in a humid chamber, with the secondary antibody conjugated to 20 nm colloidal gold (1:100), in 1.5% PBS-BSA at RT. Afterward, the grids were washed 3 times for 10 min with 3% PBS-BSA, followed by another 3 washes with PBS pH 8.0, also for 10 min, and finally washed 3 times with Milli-Q water for 5 min. After all these stages, the grids were contrasted with uranyl acetate for 30 min and lead citrate for 5 min. Subsequently, the ultrafine sections were observed in transmission electron microscopy (Zeiss 10C). 

### 2.9. Analyses of EVs’ Content

#### 2.9.1. Sterols

Purified EVs preparations (50 μL) were dried in a Speed Vac concentrator and resuspended in chloroform/methanol in the ratio of 9:1 (*v*/*v*). The sample was centrifuged at 14,000× *g* for 5 min and the organic phase separated from the precipitate and brought to dryness under a nitrogen gas stream. The sample was then solubilized in chloroform/methanol in the ratio of 2:1 (*v*/*v*) and applied on silica plates (Si 60F254s, LiChrospher, Merck, Germany) for high-performance thin layer chromatography (HPTLC). For sterol visualization, the mixture of solvents used was hexane/acetic acid/ether in the proportion of 80:40:2 (*v*/*v*/*v*), and the revelation system consisted of FeCl_3_ (0.5 mg/mL), 5% H_2_SO_4_ and acetic acid 5%, followed by heating at 100 °C for 5 min. The sterol was identified by the presence of a red violet band using commercial ergosterol (Sigma-Aldrich, St. Louis, MO, USA) as standard migration [37]. In parallel, a quantitative analysis of sterols was performed using the fluorometric kit Amplex Red Sterol Assay Kit (Molecular Probes, Eugene, OR, USA).

#### 2.9.2. Carbohydrates

Purified EVs preparations (50 μL) were incubated with 150 μL of sulfuric acid (100%) in 96-well microtiter plates (Corning™ Costar™). Afterward, 30 μL of phenol 5% was added to the systems. The solutions were homogenized, and the plate was heated at 90 °C in a water bath for 10 min. After that, the plate was subjected to cooling in another water bath at RT for 5 min. The reading was performed at 490 nm on a SpectraMax M3 microplate reader (Molecular Devices, Sunnyvale, CA, USA). Serial dilutions of glucose initiated at a concentration of 100 μg/mL were used to construct the standard curve [45].

#### 2.9.3. Proteins

Protein quantification of EVs was determined by the method described by Lowry and co-workers [46], using bovine serum albumin (BSA) as a standard. When needed, the protein dosages were made to standardize the EVs’ concentrations to be used in the experiments [46].

### 2.10. Analyses of the Diameter and Stability of EVs

Measurement of the EVs’ sizes by dynamic light scattering (DLS) was performed with a 90Plus/BIMAS Multi Angle Particle Sizing analyzer (Brookhaven Instruments Corp., Holtsville, NY, USA) as described by Frases and colleagues [47]. The stability of the EVs in PBS was tested at different times (0 to 28 days) and at −20 °C and 4 °C. Aiming the cytotoxic and interaction assays, we additionally tested the stability of EVs in the DMEM medium for 2 days at 37 °C [47].

### 2.11. Influence of EVs on Mammalian Cells

#### 2.11.1. Mammalian Cells: Cultivation

Human lung epithelial cells (A549) and murine macrophages (RAW 264.7) were maintained in DMEM supplemented with 10% of fetal bovine serum (FBS) at 37 °C in an atmosphere with 5% CO_2_.

#### 2.11.2. Mammalian Cells: Viability

The effects of *S. apiospermum* EVs on the viability of A549 and RAW cell lineages were evaluated by 3-(4,5-dimethylthiazol-2-yl)-2,5-diphenyltetrazolium bromide (MTT) assay [48]. Firstly, mammalian cells (10^5^/mL) were allowed to adhere in 96-well plates in DMEM supplemented with 10% FBS for 24 h at 37 °C in a 5% CO_2_ atmosphere. Then, the medium was discarded, and both mammalian cells were incubated with increasing concentrations of EVs (corresponding to 0.047 to 1.5 µg/mL of protein) for 24 and 48 h in DMEM. Subsequently, the formation of formazan was measured by incubating the wells for 4 h in the dark at 37 °C with MTT (5 mg/mL in PBS). The plates were then centrifuged (300× *g*/8 min), the pellet was dissolved in dimethyl sulfoxide (DMSO) and the absorbance measured in a microplate reader at 570 nm. 

#### 2.11.3. Effects of EVs on Conidia-Host Interplays

A549 and RAW cells were pretreated or not (control) with EVs (0.375 µg/mL of protein) in DMEM for 2 and 24 h at 37 °C in a 5% CO_2_ atmosphere. After the treatment, the systems were carefully washed with DMEM. Then, *S. apiospermum* conidia was added to the 24-well tissue plates to interact with both cells for 4 h in a cell ratio of 10:1 (conidia/mammalian cell) at 37 °C in a 5% CO_2_ atmosphere. Subsequently, the non-adherent conidia were removed by washing with DMEM. The systems were fixed in Bouin and stained with Giemsa. The percentage of infected cells was determined by randomly counting at least 200 mammalian cells in each of the triplicate cover slips in bright field microscopy. The association index was obtained by multiplying the percentage of infected mammalian cells by the number of conidia per infected cell. 

### 2.12. Toxicity of EVs in Galleria mellonella Larvae

*Galleria mellonella* larvae in the final instar larval stage were selected according to similarity in size, color and weight (10–15 g). Larvae (10 per group) were inoculated with 10 µL of EVs suspensions (corresponding to 2.9, 5.8 and 11.6 μg of protein/larva, respectively) in sterile PBS. The inoculations were performed with a Hamilton syringe into the hemocoel through the last proleg, as previously described [49]. The same number of caterpillars was also inoculated with sterile PBS (EVs vehicle) in each experiment to monitor potential effects due to the physical and stress injury, and a second control group was caterpillars without any manipulation. All larvae were placed in Petri dishes and maintained in the dark at 25 °C. Larval mortality was monitored daily for 7 days. Larval death was assessed by melanization and the lack of movement in response to touch stimulation. The results represent the mean percentage survival of larvae from all assays.

### 2.13. Statistical Analyses

The experiments were performed in triplicate in three independent experimental sets. Data were analyzed statistically by means of a one-way analysis of variance (ANOVA). Survival analyses were determined using the log-rank test and the Kaplan–Meier survival curves. The significance level for the *G. mellonella* assay was analyzed by a two-way ANOVA with Bonferroni post-test. All statistical analyses were performed with GraphPad Prism software 6.0 (GraphPad Software Inc., La Jolla, CA, USA), in which *p*-values of 0.05 or less were considered statistically significant.

## 3. Results and Discussion

### 3.1. S. apiospermum Mycelia Release Immunogenic Molecules

**Figure 1:**
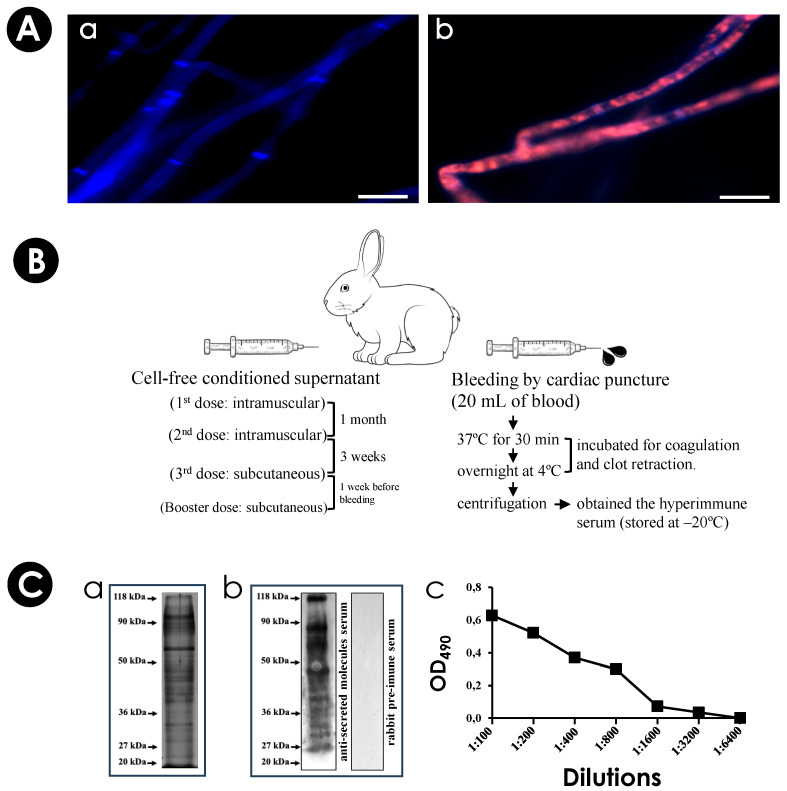
Viability of *S. apiospermum* mycelia and obtainment of hyperimmune serum. (**A**) Mycelial cells were incubated with calcofluor white and propidium iodide and analyzed by fluorescence microscopy. The left panel (**a**) shows hyphae stained only with calcofluor (in blue), indicating mycelial viability. In the right panel, hyphae killed by autoclaving have double markings with calcofluor and propidium iodide (in blue and red, respectively), indicating cell death (**b**). Bars = 10 µm. (**B**) The anti-secreted molecules serum was obtained by cardiac puncture. First, an adult white male rabbit was inoculated with the cell-free conditioned supernatant with complete Freund’s adjuvant. Two intramuscular doses were made. The third and the booster dose was subcutaneous. Bleeding was performed by cardiac puncture. After blood coagulation and clot retraction, the hyperimmune serum was separated by centrifugation and stored at −20 °C. A pre-immune serum was obtained before the rabbit received the supernatant doses. (**C**) SDS-PAGE protein profile (**a**), the reactivity of hyperimmune serum against the cell-free conditioned supernatant (at 1:400 dilution) and the no reactivity of the rabbit pre-immune serum (at 1:200 dilution) testing by Western blotting (**b**) and ELISA assay (**c**) at 490 nm (OD_490_).

Firstly, we assessed the viability of *S. apiospermum* mycelia after incubation for 24 h under chemically defined conditions (PBS–glucose medium). The results showed that mycelial cells maintained their viability, as evidenced by the absence of passive incorporation of propidium iodide, confirming the integrity of the plasma membrane (Figure 1A). Subsequently, the cell-free conditioned supernatant was obtained to check the protein profile and for inoculation into an adult male rabbit to obtain hyperimmune serum (Figure 1B). Secreted proteins were separated by SDS-PAGE (Figure 1C(a)) and through Western blotting assay (Figure 1C(b)). We showed that the hyperimmune serum recognized several antigenic molecules with molecular masses ranging from 20 to 118 kDa. Additionally, the anti-secreted molecules showed reactivity with the cell-free conditioned supernatant up to a 1:3200 dilution, as determined by ELISA (Figure 1C(c)).

**Figure 2:**
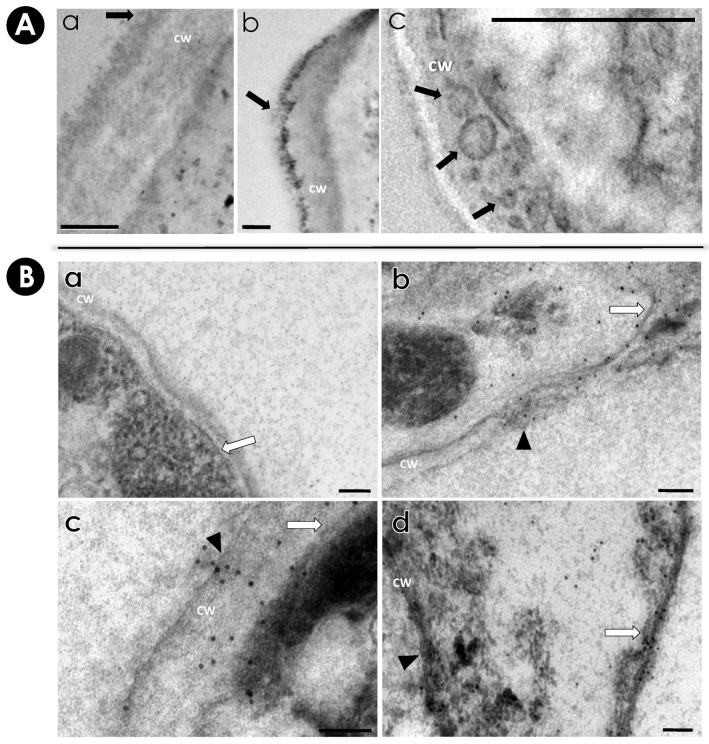
TEM and immunocytochemistry of *S. apiospermum* mycelia. (**A**) TEM images of *S. apiospermum* mycelial cells demonstrate the presence of vesicles (black arrows in a–c images) in the cell wall (CW) or released into the extracellular medium. (**B**) Mycelial cells were processed in LR WHITE hydrophilic resin and subsequently subjected to treatment for immunocytochemistry. Grids with the sections were incubated with rabbit pre-immune serum (**a**) and with anti-secreted molecules serum (**b**–**d**) at 1:20 dilution and labeled with colloidal gold (20 nm). Cell wall (CW), plasma membrane (white arrows) and EVs carrying antigenic molecules (black arrow heads) are represented. Bars = 100 µm.

TEM images revealed the presence of vesicles associated with *S. apiospermum* mycelial cells. Moreover, the vesicles were seen passing through the cell wall and released into the extracellular environment (Figure 2A). Similarly, TEM analysis of *A. infectoria*, *Candida albicans*, *Sporothrix brasiliensis*, *Histoplasma capsulatum* and *Cryptococcus neoformans* also revealed EVs in the cytoplasm near the plasma membrane, in the process of being secreted and inside the cell wall [22,25,37,50,51,52,53]. The immunocytochemistry of *S. apiospermum* mycelia revealed the localization of antigenic molecules reactive to the anti-secreted molecules serum. A pre-immune serum was used as a negative control (Figure 2B(a)). The colloidal gold labeling showed antigenic molecules in the cytoplasm, on the plasma membrane and on the cell wall of the mycelial cells (Figure 2B(b–d)). Interestingly, antigenic molecules were seen carried by vesicles, either in the vesicular membrane or in its lumen (Figure 2B(b–d), arrow heads), when passing through the cell wall (Figure 2B(b–d), CW). Similar events were observed in *C. neoformans*, where vesicular compartments containing glucuronoxylomannan (GXM) were recognized by a mouse monoclonal antibody to GXM, named mAb 18B7, and glucosylceramide (GlcCer), which were detected by an antibody to glucosylceramide (anti-GlcCer) [20,37]. In *Paracoccidioides brasiliensis*, vesicles carrying highly immunogenic α-galactosyl epitopes (both on the surface and in the lumen) were found distributed on the cell wall, following a punctuated confocal pattern, and inside large intracellular vacuoles [21]. 

### 3.2. Purification and Characterization of EVs from S. apiospermum Mycelial Secretions

**Figure 3:**
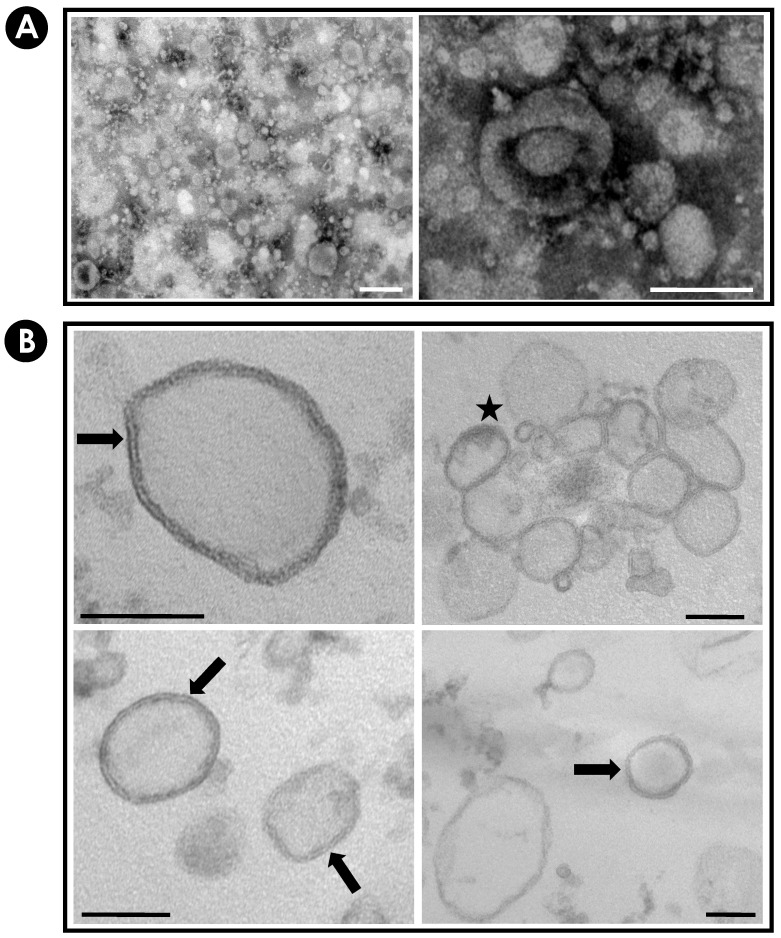
Morphological aspects of purified EVs from *S. apiospermum* mycelial cells. The images obtained by TEM confirmed the presence of EVs in the preparations. The images obtained by negative staining (**A**), as well as by ultrathin sections of the material in resin (**B**) showed vesicle populations with a high heterogeneous profile as demonstrated by morphology, diameter and electron density. Black arrows demonstrate the presence of bilayer compartments, and the star demonstrates the multivesicular bodies. Bars in (**A**) = 200 µm; Bars in (**B**) = 100 µm.

The images obtained by negative staining (Figure 3A) and by ultrathin sections (Figure 3B) confirmed the presence of purified EVs released from *S. apiospermum* mycelial cells. Vesicle populations had heterogeneous profiles in terms of morphology, diameter and electron density. The electron density of the EVs varied considerably, suggesting distinct contents. The micrographs also showed that the majority of EVs presented spherical or ovoid shape with lipid bilayer, sometimes resembling multivesicular compartments (Figure 3B) similar to those described for several fungi pathogens: *C. neoformans*, *H. capsulatum*, *Candida parapsilosis*, *C. albicans*, *Sporothrix schenckii; Saccharomyces cerevisiae*, *P. brasiliensis*, *Malassezia sympodialis*, *A. infectoria*, *Paracoccidioides lutzii*, *S. brasiliensis*, *Cryptococcus gattii*, *R. delemar*, *T. interdigitale*, *A. fumigatus*, *A. flavus* and *Candida auris* [21,23,25,26,27,28,29,30,31,32,33,34,35,37,50,53,54,55,56].

**Figure 4:**
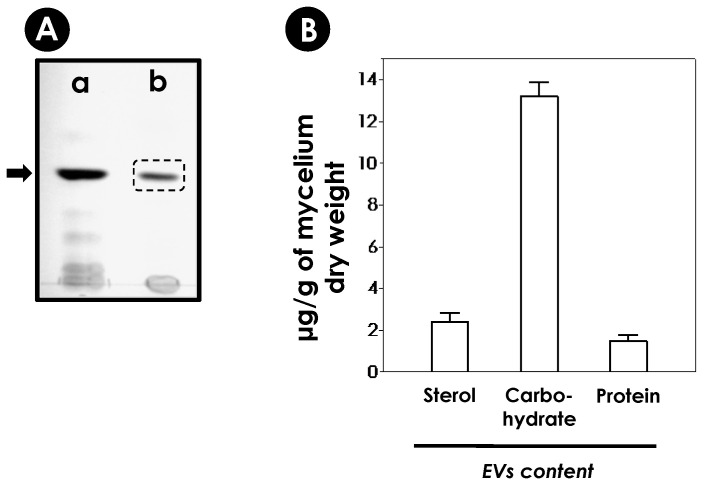
Identification and quantification of *S. apiospermum* EVs’ components. (**A**) EVs preparations obtained from *S. apiospermum* mycelia were dried and processed for HPTLC. The sterol was identified using commercial ergosterol (a, black bar) as standard migration. The dotted box in lane (b) demonstrates the sterol of fungal EVs. (**B**) Quantification of carbohydrate, protein and sterol content of the total EVs released by *S. apiospermum* mycelial cells. The results are expressed in micrograms (µg) of each vesicle content per gram (µg) of mycelium dry weight (inset) obtained from 1000 mL of culture. The results are the mean ± standard deviation (SD) of several preparations.

It is well-known that sterols are structural components of fungal EVs [37]; for this reason, these molecules were used as molecular markers of indirect identification of vesicular secretion (Figure 4A). In addition, the *S. apiospermum* mycelia obtained in each preparation (1000 mL) was dried and weighed. The total mycelial mass was 2.68 ± 0.07 g. Figure 4B shows the correlation among mycelium dry weight and carbohydrate (13.20 ± 0.67 µg/g of mycelium), protein (1.50 ± 0.29 µg/g) and sterol (2.39 ± 0.44 µg/g) content in the EVs produced by the *S. apiospermum*. 

### 3.3. Determination of the Diameter and Stability of EV

**Figure 5:**
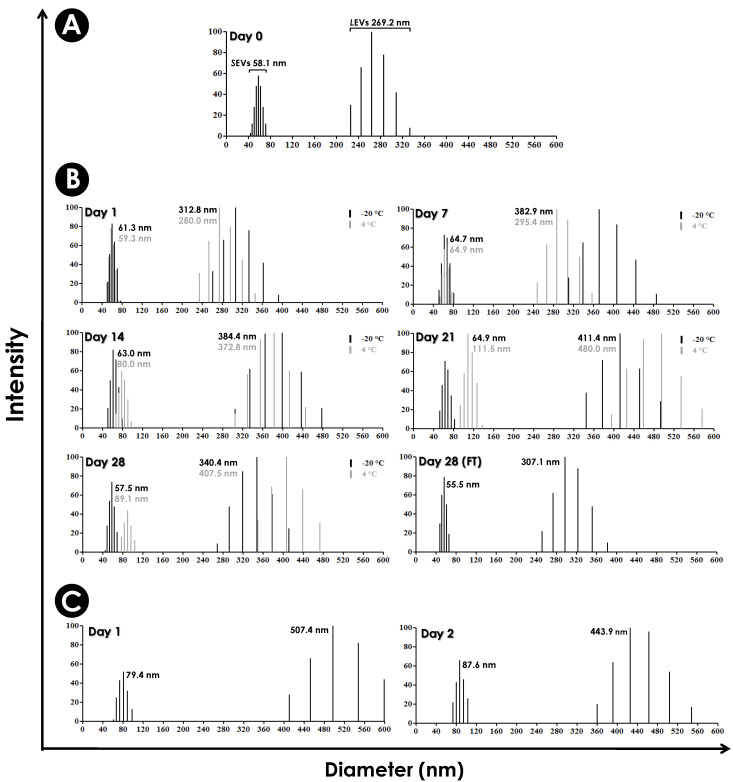
Determination of diameter and stability of *S. apiospermum* EVs. (**A**) Dynamic Light Scattering (DLS) analysis on of the effective diameter of *S. apiospermum* EVs freshly obtained in PBS, demonstrating the existence of two heterogeneously profiles: small (*S*EVs) and larger (*L*EVs) populations of vesicles. (**B**) DLS analysis of the stability of EVs in PBS stored at -20 °C and 4 °C. EVs stored at both temperatures were subjected to freezing and thawing on the days of readings (1, 7, 14, 21 and 28). Alternatively, EVs stored at -20 °C were thawed only on day 28 (freshly thawed, FT). In all conditions tested, it was still possible to observe the presence of two distinct populations. (**C**) DLS analysis of the stability of extracellular vesicles in DMEM at 37 °C. EVs incubated at 37 °C were monitored for 24 and 48 h. The average diameters of the *S*EVs and *L*EVs populations in all conditions are expressed in the graphs along with their respective intensity peaks.

By applying the DLS technique, the measurement of the effective diameter of EVs freshly obtained from *S. apiospermum* mycelial cells showed two main populations with sizes ranging from 42.9 to 65.1 nm (small EVs, *S*EVs) and from 192.8 to 275.0 nm (larger EVs, *L*EVs). The average diameters of the *S*EVs and *L*EVs populations were calculated as 58.1 nm and 269.2 nm, respectively. The total EVs mean size was calculated as 179.7 nm (Figure 5A). 

To assess the stability of the *S. apiospermum* EVs, fresh preparations were stored at −20 °C and 4 °C for 28 consecutive days. Under all tested experimental conditions, it was possible to evidence the presence of two distinct EV populations. *S*EVs and *L*EVs populations stored at 4 °C practically did not change in size until day 7. From day 14 onwards, both populations increased in size compared to day 0 (freshly obtained EVs, Figure 5A,B—peaks in gray). The EVs stored at 4 °C presented mean total population diameter of 186.1 nm (day 1), 205.9 nm (day 7), 265.8 nm (day 14), 304.5 nm (day 21) and 309.9 nm (day 28) (Figure 5B). When stored at −20 °C and subjected to freezing and thawing on reading days, there was no increase in size in the *S*EVs population, while the *L*EVs population increased significantly in size (Figure 5B—black peaks). The average diameter of the total sample population stored at −20 °C was 201.5 nm (day 1), 244.8 nm (day 7), 244.4 nm (day 14), 257.6 nm (day 21) and 224.7 nm (day 28) (Figure 5B). In parallel, EVs stored at −20 °C and thawed only on day 28 (freshly thawed EVs—FT) had a stable *S*EV population, showing an average diameter of 55.5 nm when compared to the *S*EV population at day 0 (58.1 nm). Under these same conditions, the *L*EV population showed a slight increase in mean diameter (14%) when compared to day 0. Interestingly, the mean diameter of the total EV population under these conditions (stored at −20 °C for 28 days without thawing) was 201.7 nm, exactly equal to the total mean diameter of the EVs stored at −20 °C on day 1 (201.5 nm) (Figure 5B). We also access the integrity of the EVs in DMEM medium. DMEM was used for interaction tests, which occurred for up to 48 h at 37 °C. Under these conditions, both populations showed an increase in mean diameter when compared to fresh EV populations. The mean diameter of the EV populations stored at this temperature was 366.5 nm (Day 1) and 313.7 nm (Day 2) (Figure 5C).

In *A. infectoria*, two populations of EVs were also detected, most of them ranging at approximately 50 nm [25]. On the other hand, in human pathogenic fungi, the populations of EVs are very different in diameter, with EV structures ranging from 7 to 850 nm [22,32,35,50,53,55,57,58]. The heterogeneous profile of fungi EVs is due to the distinct molecular content and the different pathways of biogenesis [58,59,60,61]. According to the biosynthetic pathway, mammalian EVs can be classified into two groups. One of them would be microvesicles, which normally have a larger size ranging from 50 to 2000 nm. These EVs are originated by budding from the plasma membrane. The second group would be exosomes, ranging from 30 to 150 nm in diameter, formed from the fusion of multivesicular bodies with the plasma membrane, with the release of the vesicles into the periplasmic space. However, the mechanisms necessary for the biogenesis of fungal EVs remain unclear, being a field full of unanswered questions. It is believed that there are several secretory pathways, whether conventional or unconventional, which together constitute a complex mechanism for the formation and release of EVs [14].

### 3.4. Detection of Immunogenic Molecules in EVs

**Figure 6:**
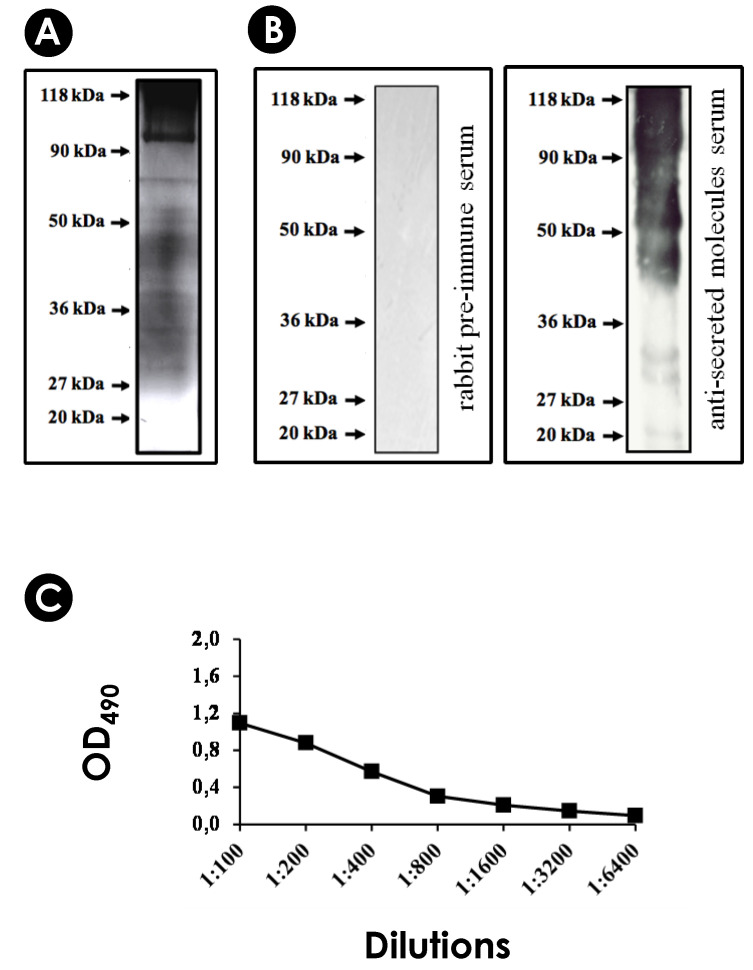
Immunogenic profile of *S. apiospermum* EVs. (**A**) Protein profile of *S. apiospermum* EVs detected by SDS-PAGE. A final concentration corresponding to 35 µg of vesicle protein content was applied to the gel. The gel was stained with silver nitrate. (**B**) Detection of antigenic proteins within *S. apiospermum* EVs by Western blotting. After the electrophoretic run, vesicular protein contents were transferred to nitrocellulose membranes, which were incubation for 1 h with the rabbit pre-immune serum (1:200) (control) or with the anti-secreted molecules serum of *S. apiospermum* (1:400), after with a secondary antibody conjugated to peroxidase and then revealed by the ECL system. The molecular weights were expressed in kilodaltons (kDa). (**C**) Immunological reactivity analysis of *S. apiospermum* EVs by ELISA assay. The reactivity of anti-secreted molecule serum was tested in several dilutions (1:100 to 1:6400) with 20 µg/mL of EVs. Negative controls with the rabbit pre-immune serum and secondary antibody conjugated to peroxidase (anti-rabbit IgG and anti-mouse IgG) demonstrated no reactivity. Molecular masses for standard proteins are indicated. (OD = Optical density).

Many research groups have demonstrated that EVs carry several immunogenic molecules that are involved with pathogenesis, virulence, metabolic pathways, transport, signaling and stress oxidation [25,62]. EVs from several fungal pathogens carry proteins that are immunoreactive with sera from human patients with histoplasmosis [50], cryptococcosis [20], paracoccidioidomycosis [21] and candidiasis [63,64] or with sera from previously infected mice with *S. brasiliensis* and *A. fumigatus* [35,53], suggesting that EVs play an important role in the adaptive immune response. By SDS-PAGE, we were able to observe the protein content profile of *S. apiospermum* EVs, showing proteins with molecular masses ranging from 20 to 118 kDa, detected by silver nitrate staining (Figure 6A). Therefore, by Western blotting, antigenic molecules of different molecular masses (from 20 to 118 kDa) were recognized by this serum, corroborating the presence of immunogenic molecules being carried by EVs (Figure 6B). Finally, the same anti-secreted molecules were used to test the global immunoreactivity of EVs through ELISA assays and a high reactivity of its content was revealed up to 1:3200 dilution (Figure 6C).

The study of fungal secretome is an important approach for the discovery of unknown virulence attributes and therefore understanding their pathogenicity. Our group has previously shown that *S. apiospermum* mycelial cells were able to actively secrete proteins into the extracellular environment; some of them were recognized by antibodies present in the serum of a human patient with scedosporiosis [17]. Another study based on immunoproteomics was developed to identify significant proteins for the diagnosis, therapy or prophylaxis of opportunistic infections caused by *Lomentospora prolificans*. The proteomic study, which utilized serum from infected mice to identify the most immunoreactive proteins in the *L. prolificans* supernatant, revealed high cross-reactivity between *Scedosporium*/*Lomentospora* species and selected several *L. prolificans* antigens that could be considered potential candidates for use in diagnosis, as therapeutic targets, and in vaccine production [65]. Although both studies were made with the total mycelial supernatant, many of the proteins identified were possibly carried by vesicles to the external environment. Indeed, proteomic approaches of EVs from *H. capsulatum*, *C. neoformans*, *P. brasiliensis*, *C. albicans*, *S. brasiliensis* and *A. fumigatus* have detected the presence of proteins involved in metabolic pathways and immunological and pathogenic activities, such as proteins involved in cell signaling, on cell growth/division, in ribosomal and proteasome function, in amino acid/protein, lipid and sugar metabolism and on anti-oxidant mechanisms [20,22,35,50,53,63,66].

### 3.5. Influence of EVs on the Interaction of S. apiospermum Conidia with Host Cells

Infections caused by *Scedosporium* species start with inhalation or inoculation of conidia, which adheres and germinates in the lung tissue; so, the interaction between fungal-mammalian cells is a crucial event for scedosporiosis development [67]. In vitro and in vivo data demonstrate that *Scedosporium* cells are able to interact with different mammalian cell lineages, such as larynx carcinoma cells (HEp2), lung fibroblasts (MRC-5), lung epithelial cells (A549), macrophages (RAW 264.7) and among others [36,68,69,70,71,72,73]. In general, the interaction event starts with the adhesion of conidia to host cells, with posterior fungal internalization; processes that are partly mediated by the peptideopolysaccharide peptidorhamnomannan (PRM) present in the fungal cell wall [36,69]. After a few hours (4 h), conidia adhered to or within the host cell germinate and caused irreversible damage by piercing the mammalian cell membrane [36,70,72]. Another study investigated the experiments on the interaction between *L. prolificans* and microglial cells (BV-2 microglial cell line), demonstrating that phagocytosis by these cells is inefficient against this fungus but is efficient with other fungal species, which are related pathogens, such as *Scedosporium boydii* and *S. aurantiacum*. Additionally, the fungus showed an increase in the percentage of hyphal branching when in contact with BV-2 cells [74]. Furthermore, molecules secreted by *Scedosporium* species are also responsible for tissue damage, as demonstrated with A549 cells co-incubated with *Scedosporium aurantiacum* and *S. apiospermum* supernatants [17,75]. 

**Figure 7:**
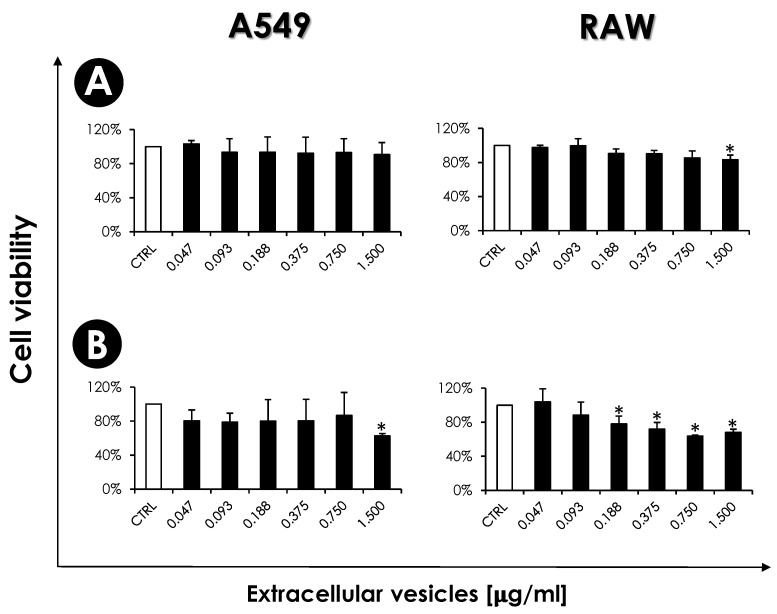
Cytotoxicity of *S. apiospermum* EVs on RAW macrophages and A549 epithelial cells. Initially, the mammalian cells (10^5^ cells) were incubated in a 96-well plate for 24 h (**A**) and 48 h (**B**) in the absence (white bars) or in the presence of the EVs at different protein concentrations (as indicated) (black bars). After that, the viability of both cells was determined spectrophotometrically at 570 nm (ABS, absorbance) by MTT assay. Data shown are the mean ± standard deviation (SD) of three independent experiments performed in triplicate. The asterisks represent the significant differences in relation to control (*p* < 0.05).

Secreted molecules play essential roles during interaction and fungal pathogenesis processes. Although the EVs obtained in our research did not originate from fungal growth at 37 °C, we acknowledge this limitation in our study. It is evident that the type and quantity of EVs produced by *S. apiospermum* at 37 °C may vary significantly, potentially impacting fungi-host interactions differently. However, this limitation does not diminish the significance of the EVs characterized in this study, which have been demonstrated to play a role in virulence, as evidenced by the in vitro and in vivo experiments conducted herein. In fact, prior to this study, little to nothing was known about the role of EVs in the interaction events of *S. apiospermum* with the host, which is precisely why we decided to investigate it. We choose the A549, as a representative cell related to the primary site of infection and RAW 264.7, as a representative phagocytic cell of the innate immune system, to start exploring this research topic. Initially, the cytotoxicity of EVs on mammalian cells was evaluated. After 24 h, no significant toxic effect was observed in A549 cells, and in macrophage cells, only the higher concentration (1.5 µg/mL) promoted significant toxic effect (Figure 7A). Also, after 48 h, the A549 cells were demonstrated to be less susceptible to the EVs than the RAW cells because only the higher concentration significantly reduced the cell viability (Figure 7B).

**Figure 8:**
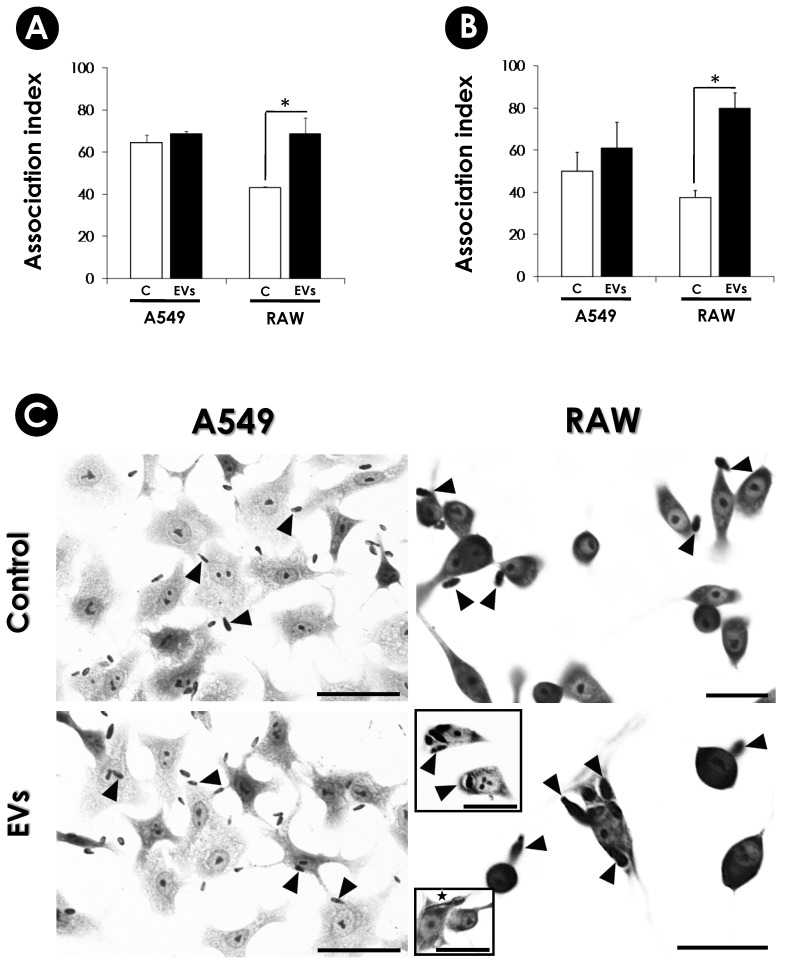
Effects of *S. apiospermum* EVs on the interaction of fungal conidia with A549 and RAW cells. A549 and RAW cells (10^5^) were pre-treated or not (control) with EVs for 2 h (**A**) and 24 h (**B**). The association index was determined by light microscopy, counting at least 200 cells in each triplicate coverslip. Data shown are the mean ± standard deviation (SD) of three independent experiments performed in triplicate. The asterisks represent the significant differences in relation to control (*p* < 0.05). (**C**) Bright field microscopy of cells pre-treated with EVs for 24 h. Black arrows point to conidia. Star points to germinated conidia, a condition usually observed in all systems with both cells. For A549 cells, bars represent 50 μm. For RAW cells, bars represent 20 μm. The images are a representative set of three independent experiments.

Subsequently, A549 and RAW cells were pre-treated with EVs (0.375 µg/mL) for 2 and 24 h, followed by the interaction with conidia for an additional 4 h. In systems pre-treated for 2 h, the association index of the fungus with the A549 cells was the same as the control well and was a significant increase (*ca*. 60%) in the interaction of conidia with RAW macrophages (Figure 8A). In 24 h, the pre-treatment of A549 cells with EVs induced some increase in the interaction with conidia, but it was not significant. However, with RAW macrophages, EVs induced a significant increase (2.2-fold increase) in the interaction of conidia when compared to the control (Figure 8B,C).

Even if it is assumed that EVs are released by fungi during the infection process, their biological roles would still be difficult to address because few studies are available. To date, some of these works have demonstrated that EVs from *C. neoformans*, *C. gattii* and *C albicans* are taken up by phagocytic cells, such as RAW and J774 macrophage lineages and bone marrow-derived (BMD) (macrophages and dendritic) cells [22,35,55,57]. Also, *C. neoformans* EVs are taken up by human brain microvascular endothelial cells (HBMEC) [76]. The EVs uptake occurs rapidly (from 15 min to 2 h), through redistribution of membrane lipid raft components and/or without fusion with plasma membranes. 

Our results demonstrated that the augmentation of *S. apiospermum* conidia adherence was much more prominent in the macrophage model of the EVs-stimulated than in the A549 pulmonary model (Figure 8). However, somewhat conflicting data show different phenotypes depending on the cell model, EV origin and conditions employed. In this sense, the pre-treatment of RAW macrophages with *C. neoformans* EVs for 16 h prior to infection resulted in a slight increase in phagocytosis, followed by an enhanced microbicidal activity [57]. In another work, peritoneal macrophages were pre-treated for 24 h with EVs from *P. brasiliensis.* Remarkable, although EVs did not promote a significant augment of adhesion/internalization of yeasts, the continuation of cell-fungi co-cultivation for an additional 48 h revealed a high fungicidal activity (*ca.* 91% of mortality) [77]. Furthermore, the EVs from *T. interdigitale* enhanced the fungicidal activity of BMD macrophages, probably by a huge proinflammatory induction [32]. The stimuli with EVs from *A. fumigatus* augment the phagocytosis of conidia by RAW macrophages (*ca.* 12%) and BMD neutrophils (*ca.* 17%). The presence of EVs enhanced the fungal clearance of both cells by 50% and 22%, respectively [35]. On the other hand, EVs from *C. gattii* were demonstrated to stimulate the survival and proliferation of the fungi in J774 macrophages [55]. Similarly, the EVs isolated from *S. brasiliensis* increased phagocytosis of BMD dendritic cells after stimulation for 30 min. However, after 24 h, a higher colony-forming unit (CFU) was obtained from those cells [53]. In an interesting work performed by Huang and colleagues [76], the pre-treatment (for 3 h) of the non-phagocytic HBMEC with *C. neoformans* EVs enhanced drastically adherence of the yeasts (4.6-fold increase) and duplicated the crossing through the tight junctions of HBMEC monolayer, which suggests that EVs enhance the invasion of *C. neoformans* across the blood-brain barrier. In fact, EVs also enhanced C. *neoformans* infection of the brain, found in both infected brains and cerebrospinal fluid of C57LB/6 mice [75]. 

Regarding *S. apiospermum*, the enhancement of fungi adhesion/internalization in the RAW model (Figure 8) may be in part due to the components delivered by the EVs and/or due to the phagocytic properties of activated macrophages after EV stimuli. However, we think that macrophages could not overcome the infection since in a few hours, conidia began to germinate, turning into long hyphae that lyse the mammalian cells by actively penetrating [70] (Figure 8C-star). In A549 cells, no alteration in fungi index was observed after EV contact (Figure 8). The reasons why this did not occur deserve further studies. Perhaps A549 cells could prevent EV endocytosis in part by their ability to secrete a mucus-like substance [70], a secretory particular property of these pulmonary cells as a pneumocyte type II [78]. In fact, no cytotoxic effects were seen in A549 cells that were EV-treated (Figure 7). Finally, different proteomic, serological and enzymatic approaches demonstrated a high compositional divergence of EVs, which some of the carried molecules may be involved in the adhesion/internalization process [20,22,35,50,53,63,66]. Therefore, the different data discussed above reflect how complex the mechanisms surrounding the EVs are.

### 3.6. Toxicity of EVs on In Vivo G. mellonella Larvae Model

**Figure 9:**
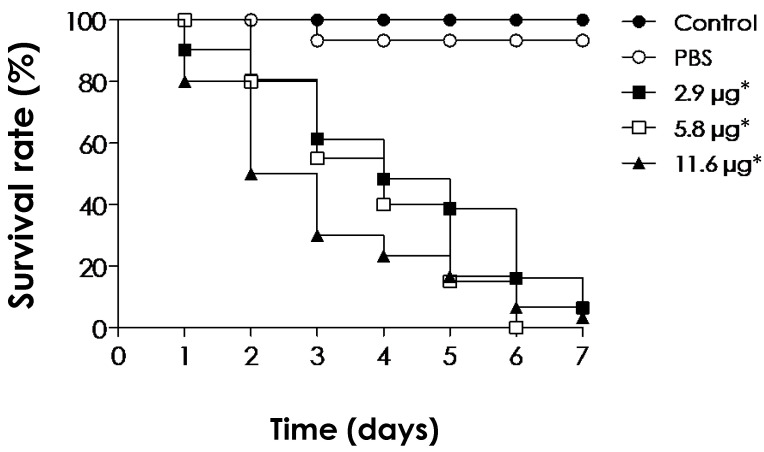
*S. apiospermum* EVs toxicity to *G. mellonella* larvae model. The toxicity was determined by inoculation of 10 µL of EVs suspensions (2.9, 5.8 and 11.6 μg/insect) in each group of 10 larvae. Controls of larvae inoculated with PBS (vehicle) or without any manipulation were also made. Larva mortality was monitored for 7 days by observing the lack of movement and melanization. Data shown are the mean of three independent experiments. Significant differences were observed between untreated (control) and EVs’ injected larvae (* *p* < 0.0001); Log-rank, ManteleCox test.

EVs isolated from *C. albicans* at higher concentrations (sterol contents corresponding to 2 μM) did not cause acute lethality in *G. mellonella* larvae [22]. Similarly, *C. neoformans* EVs also induced protection in this organism after challenge with yeast cells [79]. We, therefore, also screened the *S. apiospermum* EVs toxicity against *G. mellonella* larvae. The EVs were administered at 2.9, 5.8 and 11.6 μg of protein per insect (at day 0) and the viability of the larvae was assessed daily for 7 days. Surprisingly, all concentrations tested exhibited highly toxic effects toward the larvae, which displayed a lack of movement and melanization of their cuticle, which is indicative of an inflammatory immune response (Figure 9). The larvae mortality was shown to be dependent on the EV concentration and the time of contact. At day 7, all tested concentrations induced survival rates of less than 10% (Figure 9). The reason why the EVs induced such toxic effects is probably due to their immunogenic content, which may induce a high response by the larvae’s innate immune system. Although the roles of fungal EVs in relation to hosts are poorly understood, EVs, or outer membrane vesicles (OMVs), of Gram-negative bacteria play important roles in their pathogenicity [80,81,82]. For instance, *Campylobacter jejuni* EVs have been shown to exhibit high toxicity to *G. mellonella* larvae models [83,84]. The cytolethal distending toxin is demonstrated to be secreted within *C. jejuni* EVs, which has been shown to be, in parts, responsible for the toxic effects in *G. mellonella* larvae [83,85,86].

## 4. Conclusions

Collectively, the present work showed for the first time the presence of EVs from *S. apiospermum*. The EVs released by the mycelial form of *S. apiospermum* have a heterogeneous profile with diversified morphology, diameter and electron density, as well as contain sterol, carbohydrates, proteins and a wide range of antigenic molecules. EVs increased the interaction process with macrophages, showed cytotoxic effects in in vitro tests and were potentially toxic in the tested conditions with the in vivo *G. mellonella* larvae model. The present data highlight the importance of the study of molecules secreted (in EVs or not) by filamentous fungi and contribute to the tentative unveiling of mechanisms involved in the infectious process caused by *S. apiospermum* in scedosporiosis.

## Data Availability

Data are contained within the article.
